# Exclusive Breastfeeding Is Not Ensuring an Adequate Vitamin B Status in Premature Infants with Very Low Birth Weight

**DOI:** 10.3390/nu18030423

**Published:** 2026-01-27

**Authors:** Anne-Lise Bjørke-Monsen, Ingrid Kristin Torsvik, Mariann Haavik Lysfjord Bentsen, Thomas Halvorsen, Per Magne Ueland

**Affiliations:** 1 Laboratory of Medical Biochemistry, Førde Hospital Trust, 6807 Førde, Norway; 2 Department of Paediatric and Adolescent Medicine, Haukeland University Hospital, 5021 Bergen, Norway; ingrid.kristin.torsvik@helse-bergen.no (I.K.T.); mariann.haavik.lysfjord.bentsen@helse-bergen.no (M.H.L.B.); thomas.halvorsen@helse-bergen.no (T.H.); 3 Faculty of Medicine, Department of Clinical Science, University of Bergen, 5007 Bergen, Norway; 4 Bevital, 5009 Bergen, Norway; per.ueland@bevital.no

**Keywords:** premature, birth weight, infants, breastfeeding, vitamin B12, vitamin B2, vitamin B6, folate, homocysteine, methylmalonic acid

## Abstract

**Background**: Exclusive breastfeeding for the first 6 months of corrected age (CA) is recommended for premature infants with very low birth weight (<1500 g) (VLBW). B vitamins are essential for normal development and growth, including DNA methylation, and we investigated whether exclusive breastfeeding for the first 6 months provides an adequate cobalamin, folate, vitamin B6, and vitamin B2 status compared to additional or exclusive formula feeding from term to 12 months CA. **Methods**: In recruited infants born prematurely with VLBW, levels of vitamin B12 (cobalamin), folate, vitamin B6 (pyridoxal 5′-phosphate, PLP), vitamin B2 (riboflavin), and the metabolic markers, total homocysteine (tHcy) and methylmalonic acid (MMA), were determined at term (*n* = 35) and at 2 (*n* = 47), 6 (*n* = 48), and 12 months (*n* = 58) CA. **Results**: Only a minority of the infants were given multivitamin supplementation, and this was associated with higher PLP and riboflavin levels. One-third of the infants were exclusively breastfed to 6 months CA, and these had lower cobalamin, PLP, and riboflavin concentrations compared to formula-fed infants; additionally, 80% had plasma tHcy concentrations ≥ 6.5 µmol/L, indicative of cobalamin deficiency during the first 6 months CA. Serious deficiency of one or more B vitamins was evident in 3–9% of the infants at each time point during the first 12 months CA, more often in breastfed infants, but not exclusively. **Conclusions**: Exclusive breastfeeding with inadequate multivitamin supplementation and no specific recommendation for introduction of solid food does not provide an adequate B vitamin status in infants born prematurely with VLBW. Nutritional micronutrient recommendations must be improved, and regular evaluation of vitamin status should be implemented in the follow-up for premature VLBW infants.

## 1. Introduction

Infancy is a period of rapid growth and development, and an adequate micronutrient status is important for optimal growth and development, particularly of the central nervous system [1]. Infant micronutrient status depends on gestational age (GA) and birth weight (BW), in addition to maternal micronutrient status during pregnancy and also after delivery for those who are exclusively breastfed [2]. Infants born premature and with very low birth weight (VLBW) (<1500 g) have low vitamin stores and accelerated “catch-up” growth, which requires an adequate amount of energy and essential nutrients, including vitamins [3].

Human milk is considered the optimal nutrition source for all infants, including VLBW infants [4]. As VLBW infants initially only tolerate low feeding milk volumes, the nutritional requirements cannot be met with human milk alone, and multinutrient fortifiers are added to hospitalized VLBW infants [5]. Most of the available commercial fortifiers contain varying amounts of protein, carbohydrate, calcium, phosphate, and other minerals, as well as all the vitamins and relevant trace elements [6,7]. There is currently no consensus about the need for post-discharge fortification to optimize growth and neurodevelopment among VLBW infants fed human milk [7].

Breastfeeding has several benefits for newborn infants [8], including numerous immunological and bioactive factors [9], and for this reason, breast milk represents the gold standard for every infant, including the premature [10,11]. However, human milk is low in vitamin K1 and D [12,13], and there has been concern about additional low levels of vitamin B12 (cobalamin), vitamin B6 (pyridoxal 5′-phosphate, PLP), and vitamin B2 (riboflavin) [14]. The B vitamin concentrations in human milk depend on maternal B vitamin status and lactational stage [15]. Most B vitamin concentrations in human milk, apart from folate, which remains high, decrease over the first 2–4 months of lactation [15]. Maternal deficiency is reflected in low vitamin concentrations in the milk and an increased risk for infant B vitamin deficiency with extended periods of exclusive breastfeeding [14,16].

As decision limits for deficiency are missing for many B vitamins, evaluation of B vitamin status may be difficult in this age group. A serum cobalamin concentration below 400 pmol/L [17,18] and a plasma total homocysteine (tHcy) concentration of 6.5 µmol/L has been suggested as decision limits for cobalamin deficiency in infants [19]. The metabolic cobalamin marker methylmalonic acid (MMA) is inversely related to cobalamin, but in young children, MMA is considered to be inferior to plasma tHcy as a cobalamin marker [20]. Plasma tHcy is reported to increase when serum folate falls below ~25–27 nmol/L, indicating suboptimal intracellular folate stores below this level, with a steeper increase below 10 nmol/L, and a serum folate of 10 nmol/L is often used as a decision level of deficiency in adults [21], but no limits are published for infants. For plasma vitamin B6, a plasma PLP concentration below 30 nmol/L is considered to indicate vitamin B6 deficiency in adults [22], whereas a plasma PLP concentration above 50 nmol/L is reported to ensure an optimal vitamin B6 status in women of fertile age [23]. Riboflavin is a precursor for the coenzymes flavin mononucleotide (FMN) and flavin adenine dinucleotide (FAD). The given concentrations of riboflavin in plasma include FMN. The current gold-standard marker for riboflavin status is erythrocyte glutathione reductase activation coefficient (EGRac), and deficiency has been defined as EGRac ≥ 1.4 and suboptimal status as EGRac ranging from 1.2 to 1.39 [24]. In older adult women, the inflection point between EGRac and plasma riboflavin occurred at a plasma riboflavin concentration of 26.5 (95% CI: 20.5, 32.5) nmol/L and at an EGRac of 1.25 [25].

Recommendations for both water- and fat-soluble vitamin intake in premature infants are reported to be inconsistent, with wide variations [26]. A recent survey on vitamin and mineral supplementation practices in preterm infants from Australia and New Zealand showed that, while prescription of vitamin D was common and one-third also prescribed folic acid, neither cobalamin, vitamin B6, nor vitamin B2 supplementation was mentioned [27].

Cobalamin, folate, vitamin B6, and vitamin B2 are essential micronutrients important for cellular function, metabolism, DNA synthesis and methylation, psychomotor development, and growth [28]. Published articles on B vitamin status, particularly for vitamins like PLP and riboflavin, in the follow-up of premature VLBW infants are missing. A relevant question is whether the recommendations for premature VLBW infants ensure an adequate B vitamin status. The aim of the present study was, therefore, to investigate B vitamin status in relation to exclusive breastfeeding versus additional or exclusive formula feeding in premature infants with VLBW from term to 12 months CA.

## 2. Materials and Methods

### 2.1. Study Population and Design

During 2008 and 2009, 64 premature infants with a birth weight below 1500 g were recruited to an observational longitudinal study from the neonatal unit at the Department of Pediatrics, Haukeland University Hospital, Bergen, Norway. Infants who were seriously sick were excluded. Gestational age was based on ultrasonography at 17–18 weeks of gestation; corrected age (CA) was calculated as if they were born on their expected delivery date; and small for gestational age (SGA) was defined as BW less than the 10th percentile for GA according to growth trajectories for Norwegian infants [29]. Growth parameters and nutrition during their stay at the neonatal unit were recorded. After discharge, all infants and mothers were invited back for follow-up at CA term and at 2, 6, and 12 months, but not all infants attended all time points. At each visit, a non-validated questionnaire on infant and maternal nutrition and use of micronutrient supplementation was completed, infant growth parameters were measured, and blood samples were collected from the infant and the mother.

Ethical approval of the protocol was granted by the Regional Committee for Medical Research Ethics, Western Norway (104.08). All procedures were performed in accordance with the Declaration of Helsinki, and written informed consent was obtained from all the mothers.

### 2.2. Nutrition

#### 2.2.1. Diet

All participants received nutrition according to current procedures in our neonatal unit during their stay. When enteral nutrition was fully established, human milk was enriched with human milk fortifier containing protein, vitamins, and minerals, while infant formula was enriched by adding 2.5 g of extra powder per 100 mL milk. Fortification of human milk was continued until a weight of approximately 2 kg.

Post-discharge nutritional recommendations included exclusive breastfeeding to 6 months CA. Random assignment to nutrition/monitoring groups was not performed, as it was left to the mother’s preference to exclusively breastfeed or to administer supplemental or exclusive formula. Introduction of solid food, usually provided as infant cereals that contained 15–45 µg folic acid, 0.09–0.3 mg vitamin B6, and 3–10 mg iron per 100 g powder, was recommended to start thereafter.

#### 2.2.2. Multivitamin Supplementation

When the infants received 100% enteral feeding or tolerated at least 100 mL/kg/day, additional multivitamin supplements were prescribed. Daily folic acid intake of 0.1 mg (Apotek, Oslo, Norway) was recommended up to 3 months. Additional multivitamins were given as Multibionta (Merck Selbstmedikation GmbH, Darmstadt, Germany) daily to 3 weeks, and then replaced by Sanasol or Biovit (both Orkla Health AS, Oslo, Norway), 5 mL twice a day to 6 months CA. While Multibionta contained 1.2 mg vitamin B12 per 5 mL, neither Sanasol nor Biovit contained this vitamin, but all contained vitamin B2, vitamin B6, and folate.

Vitamin D drops (10 µg per day) or cod liver oil 5 mL were recommended daily from 3 weeks of age throughout the first year [30], and iron was recommended as ferrous fumarate mixture (Nycomed Pharma AS, Asker, Norway), 9 mg daily from 6 weeks to 6 months, and 18 mg daily to 12 months of age. This regimen was applied whether they were breastfed or received formula; however, 50% lower amounts of multivitamins were given to the formula group.

### 2.3. Blood Sampling and Analyses

Blood samples for preparation of EDTA–plasma were placed in ice water, and plasma was separated within 4 h. The samples were stored at −80 °C for two years until analysis.

Serum cobalamin was determined by a Lactobacillus leichmannii microbiological assay [31], and serum folate by a Lactobacillus casei microbiological assay [32]. Vitamin B2 exists in blood as riboflavin and its cofactor FMN [24]. The active form of vitamin B6 in blood is PLP [33]. B2 and B6 vitamers were analyzed using an LC-MS/MS assay [34].

Plasma tHcy and serum MMA were assayed using a (GC-MS) method based on methylchloroformate derivatization [35]. Analyses of vitamins and biomarkers were carried out at BEVITAL AS (www.bevital.no).

### 2.4. Statistical Analysis

Means and standard deviations (SD) and median and interquartile ranges (IQR) were calculated for normally distributed and non-normally distributed data, respectively. Means and medians were compared using Student’s *t*-test and the Mann–Whitney U test, respectively. Differences in categorical variables were tested by the Chi-square test.

Multiple linear regression models were used to assess determinants of vitamin status at term age and at 6 months CA. The model included exclusive breastfeeding versus combined or exclusive formula feeding, infant and maternal use of multivitamins, gender, SGA, gestational age, and weight at birth, as well as weight at current age. At CA 6 months, infant intake of porridge and dinner per day were also included.

Graphical illustrations of the relationships between serum cobalamin and plasma tHcy—adjusted for serum folate—between serum folate and plasma tHcy—adjusted for serum cobalamin—and between plasma PLP and riboflavin in infants at term and at 2, 6, and 12 months CA were depicted as generalized additive models (GAMs).

The SPSS statistical program (version 29) (SPSS Inc., Chicago, IL, USA) and the packages “mgcv” in R, version 4.2.3. (The R Foundation for Statistical Computing, Vienna, Austria) were used for the statistical analyses. Two-sided *p*-values < 0.05 were considered statistically significant.

## 3. Results

### 3.1. Demographics and Nutrition

#### 3.1.1. Mothers

Demographic variables for the 60 mothers are presented in Table 1. The majority of the mothers were primipara, 18% (11) had one former child, and 17% (10) had two–four former children. Daily use of multivitamin supplement was reported by 31% (11/35) of the mothers at term, by 6% (3/45) at 2 months, by 26% (12/45) at 6 months, and by 18% (10/55) at 12 months CA. Four mothers (7%) reported daily smoking during pregnancy (1–10 cigarettes per day), and two mothers were still smoking at corrected term (2–3 cigarettes per day).

#### 3.1.2. Infants

##### During the Hospital Stay

Sixty-four infants were included in this study; fourteen of these were twins. In ten twin pairs, only one of the infants was included in this study, while both twins were included in two twin pairs. The recruited 64 infants attended one or more follow-up visits, *n* = 35 at corrected term, *n* = 47 at CA 2 months, *n* = 48 at CA 6 months, and *n* = 58 at CA 12 months. Mean length of stay in the neonatal unit was 64 (SD 27) days, with a range of 21 to 120 days.

The majority of the infants (*n* = 50, 78%) received blood transfusions, with a mean number of 3 (SD 3) transfusions, range 1–13. Fourteen (22%) infants experienced intracranial bleeding, of which three of the fourteen (21%) had grade 3–4, two infants were diagnosed with periventricular leukomalacia, and two infants had necrotizing enterocolitis and received surgery. Eight of the sixty-four infants (13%) received steroids due to pulmonary problems.

From birth, all infants received either maternal milk or donated human milk with additional nutrient supplementation (Nutriprem (Nutricia, Danone, Amsterdam, The Netherlands), Minimax (Nestle, Vevey, Switzerland)), FM85/Pre NAN Human Milk Fortifier (Nestle, Vevey, Switzerland) until they reached a weight of 1500 g. From a weight of 1500 to 2000 g, 1 infant received formula (Collet^®^; Collet Marwell Hauge AS, Asker, Norway), and the remaining 63 infants were given human milk, of whom 61 received additional nutrient supplementation.

Mean weight at discharge to home was 2389 (SD 442) grams, with a range of 1700 to 3600 g.

##### After Discharge to Home

Of the 64 infants, 29 were examined at all four time points, 14 at three time points, and 21 at two time points. Growth parameters, type of nutrition, and use of supplements were recorded at term (*n* = 35) and at 2 months (*n* = 45), 6 months (*n* = 47), and 12 months CA (*n* = 58) (Table 2). The median weight increased by 204%, median length by 57%, and median head circumference by 30% from term to 12 months CA. At term, infants who were exclusively breastfed had significantly higher mean weight, 3079 (SD 389) grams, compared to infants who received additional or exclusively formula (mean weight 2822 (SD 376) grams), *p* = 0.05. No significant differences were seen for growth parameters at 2 and 6 months CA according to nutrition with human milk or formula.

Fifty-eight percent (32/55) of the infants received exclusive breastfeeding for a mean of 1.9 (SD 2.0) months, with a range of 0.5 to 6.5 months, while the remaining 23 infants were given either mixed feeding or formula feeding. Mean age at introduction of porridge was 5.9 months (SD 1.3), ranging from 3 to 9.5 months CA. Mean age at introduction of dinner was 7.2 (SD 1.3) months, ranging from 4.5 to 10 months CA.

All infants received folic acid supplementation at 2 months CA, and iron supplementation was stopped for all infants at 12 months. Almost half of the infants received micronutrient supplements at term, but only a minority received these from 2 to 12 months CA (Table 2).

### 3.2. Infant B Vitamin Status

Infant B vitamin status at term and at 2, 6, and 12 months CA is shown in Table 3. During this period, median serum cobalamin increased by 44%, while serum folate decreased by 81%, PLP by 44%, and riboflavin by 47%, and FMN increased by 51%.

**Figure 1:**
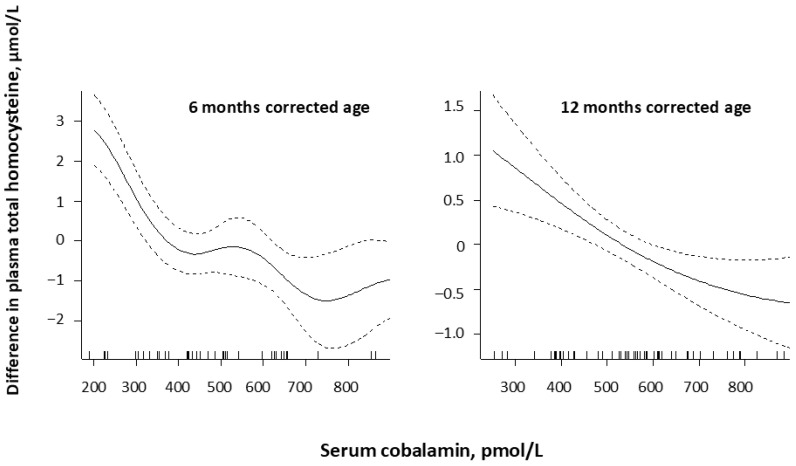
Serum cobalamin in relation to plasma total homocysteine concentrations corrected for serum folate by generalized additive models (GAMs) in premature infants at 6 (*n* = 48) and 12 (*n* = 58) months corrected age. The values on the y-axes are given as differences from the respective mean values. The dashed lines around the solid GAM curve shows the 95% CI around the central estimate.

Plasma total homocysteine (tHcy) was significantly negatively correlated with serum cobalamin at 2 months (rho: −0.49, *p* < 0.001), 6 months (rho: −0.59, *p* < 0.001), and 12 months (rho: −0.40, *p* = 0.002) CA, but not at corrected term (rho: −0.1, *p* = 0.63). At 6 and 12 months CA, plasma tHcy started to increase when serum cobalamin fell below 800 pmol/L. At 6 months CA, there was a steeper increase below 400 pmol/L (Figure 1). MMA was reduced by 65% from term to 12 months and was correlated with serum cobalamin at 2, 6, and 12 months CA (rho < −0.38, *p* < 0.001), but not at term CA.

**Figure 2:**
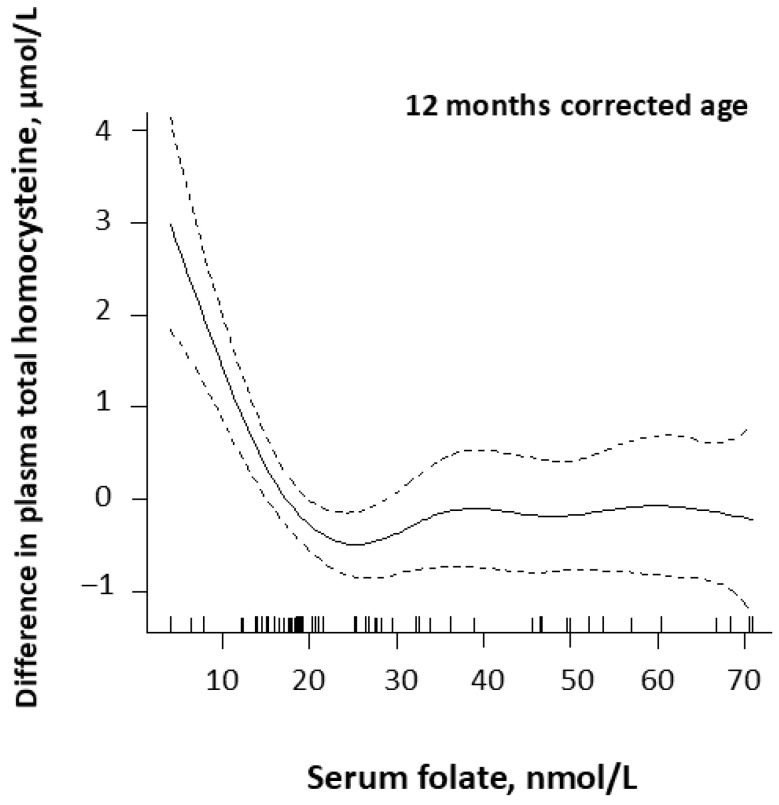
Serum folate in relation to plasma total homocysteine concentrations corrected for serum cobalamin by generalized additive models (GAMs) in premature infants (*n* = 58) at 12 months corrected age. The values on the y-axis are given as differences from the respective mean values. The dashed lines around the solid GAM curve shows the 95% CI around the central estimate.

A significant correlation between plasma tHcy and serum folate (rho: −0.27, *p* = 0.05) was only observed at 12 months CA. Figure 2 shows that plasma tHcy increased steeply when serum folate fell below 25 nmol/L at 12 months CA.

Plasma tHcy was positively correlated with plasma PLP at corrected term (rho: 0.56, *p* < 0.001), but not later, and was not correlated with plasma riboflavin at any time point (*p* > 0.12).

**Figure 3:**
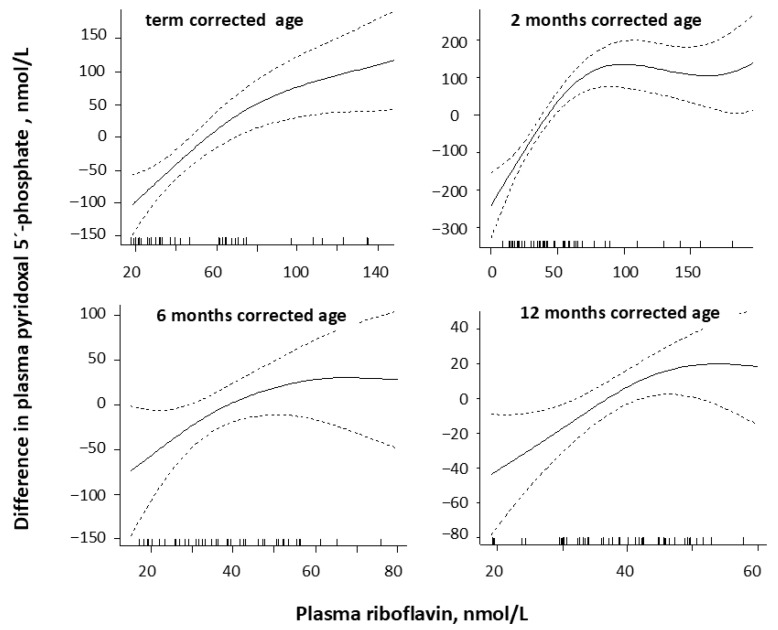
Plasma riboflavin in relation to plasma pyridoxal 5′-phosphate concentrations by generalized additive models (GAMs) in premature infants at corrected term (*n* = 35) and at 2 (*n* = 47), 6 (*n* = 48), and 12 (*n* = 58) months corrected age. The values on the y-axes are given as differences from the respective mean values. The dashed lines around the solid GAM curve shows the 95% CI around the central estimate.

Plasma PLP was significantly correlated with plasma riboflavin at term (rho = 0.68, *p* < 0.001) and at 2 (rho: 0.77, *p* < 0.001), less so at 6 (rho = 0.32, *p* = 0.03), and at 12 months (rho = 0.40, *p* = 0.002), as shown in Figure 3.

#### 3.2.1. Vitamin Deficiency

Low and severely deficient vitamin status was evident in some infants at all time points, as demonstrated by the 25th percentile and the minimum values for cobalamin, folate, PLP, and riboflavin given in Table 3. The majority of infants with vitamin deficiencies were exclusively breastfed, but low levels of folate, PLP, and riboflavin were also seen in infants who had mixed feeding or exclusive formula feeding.

Two infants were exclusively fed human milk from weight 1500 g to 6 months CA, and both had low vitamin levels at term and at 2 and 6 months CA. PLP and riboflavin levels were particularly low at term and at 2 months, while serum cobalamin in one of the infants was 48 pmol/L at 6 months.

Low cobalamin levels (<400 pmol/L) were seen in 51% (*n* = 18/35) at term, in 47% (*n* = 22/47) at 2 months, in 33% (*n* = 16/48) at 6 months, and in 19% (*n* = 11/58) at 12 months CA. Very low cobalamin levels (<148 pmol/L) were seen in two infants at 2 months, in three infants at 6 months, and in one infant at 12 months CA. The percentage of infants with a high plasma tHcy (>6.5 µmol/L)—indicative of cobalamin deficiency—was 29% at term (*n* = 10/35), 75% (*n* = 35/47) at 2 months, 42% (*n* = 20/48) at 6 months, and 10% (*n* = 6/58) at 12 months CA.

Low folate levels (<25 nmol/L) were seen in 3% (1/35) at term, in 6% (*n* = 3/47) at 2 months, in 29% (*n* = 14/48) at 6 months, and in 53% (*n* = 31/58) at 12 months CA. Very low serum folate levels (<10 nmol/L) were seen in two infants at 2 months, in one infant at 6 months, and three infants at 12 months CA.

Plasma PLP levels—indicative of deficiency in adults (PLP < 30 nmol/L)—were seen in two infants at 2 months, and in one infant at 6 and 12 months CA.

Plasma riboflavin levels below the optimal for adults (<26.5 nmol/L) were seen in 31% (*n* = 9/35) at term, in 38% (*n* = 18/47) at 2 months, in 40% (*n* = 19/48) at 6 months, and in 40% (*n* = 23/57) at 12 months CA.

#### 3.2.2. Determinants of B Vitamin Status

As Table 4 and Table 5 show, infants who were exclusively breastfed had lower levels of cobalamin, PLP, and riboflavin at term (except for cobalamin) and at 2 and 6 months CA, and had higher levels of tHcy and MMA at 6 months CA compared to infants who received formula. No differences were seen for serum folate according to feeding practice.

The strongest determinant for infant vitamin status at term and at 2 and 6 months CA was exclusive breastfeeding versus mixed feeding (breastfeeding in combination with formula) or exclusive formula feeding, in a multiple linear regression model, which additionally included gender, SGA, gestational age, weight at birth, weight at current age, and infant and maternal use of multivitamins (Table 4). Use of vitamin supplementation increased plasma PLP and riboflavin levels, but had no effect on folate and cobalamin levels at term, or at 2 and 6 months CA.

High tHcy levels > 6.5 µmol/L—indicative of cobalamin deficiency—were more common among exclusively breastfed infants compared to exclusively formula-fed infants at 2 months (82% versus 27%) and at 6 months CA (80% versus 9%).

## 4. Discussion

Exclusive breastfeeding was reported in one-third of premature VLBW infants from term to 6 months CA. Exclusively breastfed infants had lower cobalamin, PLP, and riboflavin concentrations compared to formula-fed infants, and 80% had tHcy concentrations ≥ 6.5 µmol/L—indicative of cobalamin deficiency—during the first 6 months of CA. Plasma tHcy concentrations increased when serum cobalamin fell below 400 pmol/L and serum folate fell below 25 nmol/L, indicative of vitamin deficiency. Severe deficiency of one or more B vitamins was evident in 3–9% of the infants at each time point during the first 12 months of CA. Multivitamin supplementation was used by a minority, and was associated with higher PLP and riboflavin concentrations. Apart from a higher mean weight at term in exclusively breastfed infants, no differences in growth parameters were observed according to type of nutrition up to 12 months.

B vitamins and metabolic markers differ according to age, and the highest blood vitamin concentrations in humans are typically seen in term-born infants with an adequate-for-gestational-age weight during the first months of life [20,36], when both infant stores and human milk are rich in B vitamins [14]. After the first two to three months of life, most B vitamins decrease throughout the first year of life to levels observed in older children [20], a pattern compatible with a reported reduction in B vitamin levels in human milk [14] and depletion of infant vitamin stores due to increased growth [20,36].

There are published decision limits for cobalamin status [18], but decision limits for folate, riboflavin, and vitamin B6 deficiencies are lacking in infants, particularly in infants born prematurely with VLBW. It is therefore difficult to determine an adequate vitamin status in this age group. However, during the first year of life, body weight triples [3], and clinical decision limits for vitamins in infants need to be higher than in older children and adults.

We observed that plasma tHcy gradually started to increase when serum cobalamin fell below 700 to 800 pmol/L, with a steeper increase below 400 pmol/L, indicative of deficient intracellular cobalamin status in the premature VLBW infants at 6 months CA. The same pattern has been reported in 4-day-old newborns [18].

Serum folate levels were high during the first few months of life, as reported before [20,36], but at 12 months, CA plasma tHcy increased steeply when serum folate falls below 25 nmol/L, indicating folate deficiency. Based on these observations, we suggest serum cobalamin < 400 pmol/L and serum folate < 25 nmol/L as the clinical decision limits for deficiency in infants.

In children, the active form of vitamin B6, plasma PLP, is reported to progressively decrease throughout the first year and then more slowly throughout life [37], and to be lower in breastfed compared to formula-fed infants [36]. The median PLP concentration in our formula-fed premature infants decreased by one-third from term to 6 months, whereas the median PLP concentration in exclusively breastfed infants remained low from term to 6 months, similar to infants with a slightly low birth weight (2000 g) [36]. Based on these data, it is questionable whether exclusive breastfeeding can ensure an optimal vitamin B6 status in low-birth-weight infants [38].

From term to 6 months CA, plasma riboflavin was high and decreased in formula-fed infants, while it was lower and remained stable during this period in exclusively breastfed infants. In formula-fed infants with a low birth weight (2000–3000 g), plasma riboflavin decreased from a median of 62 (IQR 43, 84) nmol/L at 6 weeks to 34 (23, 50) nmol/L at 6 months, whereas in breastfed infants, plasma riboflavin was much lower, with a median of 16 (IQR 14, 23) nmol/L at 6 weeks, and it remained fairly stable, at a median of 15 (IQR 11, 19) nmol/L, to 6 months [36]. This is analogous to the PLP pattern described above, and it is questionable whether exclusive breastfeeding can ensure an optimal riboflavin status in low-birth-weight infants [38].

Riboflavin is required for the generation of the functionally active vitamin B6 form, PLP, in tissues [39], and animal studies show that riboflavin deficiency causes a decrease in the conversion of pyridoxine to PLP, and may therefore be the limiting nutrient for maintaining an adequate vitamin B6 status [40]. In premature infants, we observed a change in PLP concentrations, with plasma riboflavin levels around 80 nmol/L at term and 2 months CA, and around 50 nmol/L at 6 and 12 months CA. Whether these breakpoints can be regarded as clinical decision limits for plasma riboflavin to ensure an optimal PLP status or merely reflect a correlation in intake of vitamins B6 and B2 is unknown.

Micronutrient concentration in human milk fluctuates with maternal factors and lactational stage, and most vitamins and trace elements decrease during the first 2–4 months [15,16]. Vitamin deficiency is more common in exclusively breastfed infants than in formula-fed infants, as formula is supplemented with several micronutrients [41,42,43,44]. In this study, one-third of the premature VLBW infants were exclusively breastfed to 6 months CA, and these infants had lower cobalamin, PLP, and riboflavin concentrations compared to formula-fed infants. Eighty percent of exclusively breastfed infants had a tHcy concentration ≥ 6.5 µmol/L, indicating cobalamin deficiency [19]. This is worrying because even moderate cobalamin deficiency (tHcy ≥ 6.5 µmol/L) in infants is associated with functional motor impairment and clinical symptoms, which can be improved by cobalamin supplementation, as demonstrated in randomized, double-blind cobalamin intervention studies in infants [36,45].

Improvements in neonatal care have increased the survival rate of preterm VLBW infants and exposed several long-term sequelae throughout life. Compared to normal-birth-weight controls, these infants have an increased risk of neurodevelopmental sequelae, poorer physical abilities and respiratory function, and a predisposition to develop metabolic syndrome and cardiovascular diseases in later life [46,47].

The data material was collected in 2008–2009, but it is still relevant, as there has not been substantial improvement in nutritional recommendations to premature VLBW infants since then. Breastfeeding is still a gold standard, also for premature infants [10], and the recommendations for vitamin intake are inconsistent and vary considerably in newly published surveys on guidelines on nutrition of preterm infants [26]. While recommendation of vitamin D and iron supplementation are common, recommendations for supplementation of other water- and fat-soluble vitamins, including B vitamins, are rare [26,27].

Dietary imbalances during fetal life and infancy may cause long-lasting epigenetic effects and alterations in the expression of genes that regulate normal brain function [48]. Deficiency of cobalamin, folate, vitamin B6, and vitamin B2 are associated with hypomethylation [49], and while DNA methylation is essential for viability in human cells, global hypomethylation has a wide spectrum of effects that include genetic, epigenetic, and metabolic alterations, and is associated with the occurrence and progression of cancer and metabolic diseases [50,51], rendering it important to secure an adequate B vitamin status during growth and development [49]. Nutritional micronutrient recommendations must be improved, and as weight gain alone is not an optimal indicator of nutritional status [47], regular evaluation of vitamin status should be implemented in the follow-up for premature VLBW infants.

### Strengths and Limitations

Premature VLBW infants constitute about 0.4% of all live births in Norway and have a higher mortality compared to term-born infants [52]. The small number of infants is a limitation to this study, as data analysis from a relatively small sample size, including cross-sectional and longitudinal data, may offer potential for bias. However, we included repeated measures of various variables, which enabled us to judge the time course of vitamin status and growth during the first 12 months of CA, and also increased statistical power [53]. It is reported that increasing the number of follow-up measures from a single one to three or four can reduce sample sizes by 35–70% [54], and this applied to 67% of the infants in our study.

Nutritional research is challenging [55], and maternal reports on infant diet may be inaccurate [56], particularly during the first year of life of premature VLBW infants, which is a limitation of this study.

There are also very few, if any, published data on post-discharge nutritional status in premature VLBW infants, particularly for vitamins like PLP and riboflavin, and this study presents accurately analyzed data on four essential B vitamins and their metabolic markers during the first year of CA, which is a strength of this study.

## 5. Conclusions

Serious deficiencies of cobalamin, folate, vitamin B6, and vitamin B2 were seen in some premature VLBW infants during the first 12 months of CA, also in formula-fed infants. Exclusively breastfed infants had significantly lower levels of cobalamin, PLP, and riboflavin and higher tHcy compared to formula-fed infants during the first 6 months of CA.

Exclusive breastfeeding for extended periods, with inadequate multivitamin supplementation and no specific recommendation for introduction of solid food, does not seem to provide an adequate B vitamin status in infants born prematurely with VLBW. Improved nutrition with regular control of B vitamin status is warranted during the early period of life in premature VLBW infants.

## Figures and Tables

**Table 1:** Demographic characteristics of mothers and premature infants with very low birth weight at birth.

**Mothers (*n* = 60)**	
Age, years, mean (SD), min–max	31 (6)
BMI before pregnancy, mean (SD)	24.9 (5.5)
Parity 0, *n* (%)	39 (65%)
Interbirth interval, months, median (IQR)	44 (29, 99)
Regular use during pregnancy, *n* (%)	
Micronutrient supplements	45 (75%)
Iron supplements	9 (15%)
**Infants (*n* = 64)**	
Gestational age, weeks, mean (SD), range	28.9 (2.3), 23.6–35.5
Birth weight, g, mean (SD), range	1066 (270), 400–1490
Birth length, cm, mean (SD), range	36 (3), 28–41
Head circumference, cm, mean (SD), range	26.2 (2.1), 21–29
Small for gestational age, *n* (%)	30 (47%)
Twins, *n* (%)	14 (22%)
Gender, boys *n* (%)	35 (55%)

**Table 2:** Growth parameters and nutrition of premature infants with very low birth weight at term and at 2, 6, and 12 months corrected age.

Parameters	Corrected Age			
	Term	2 Months	6 Months	12 Months
	*n* = 35	*n* = 47	*n* = 48	*n* = 58
Weight, g, mean (SD),	2937 (400)	4769 (758)	7202 (1308)	8933 (1385)
min, max	2172, 3695	3295, 6320	4745, 11,200	5680, 12,370
Length, cm, mean (SD),	47 (3)	55 (3)	66 (3)	74 (3)
min, max	33, 51	42, 57	58, 72	63, 81
Head circumference, cm, mean (SD),	35.5 (1.6)	39.2 (3.0)	43.5 (1.6)	46.1 (1.6)
min, max	32.0, 40.8	31.5, 57.0	40.0, 48.5	43, 51
Daily intake, number of infants (%)				
Human milk only	16 (46%)	17 (36%)	15 (31%)	5 (9%)
Human milk + formula	14 (40%)	13 (28%)	11 (23%)	2 (3%)
Formula only	5 (14%)	17 (36%)	22 (46%)	13 (22%)
Cow’s milk	0	0	0	36 (62%)
Porridge	0	8 (17%)	46 (96%)	58 (100%)
Dinner	0	1 (2%)	43 (90%)	58 (100%)
Micronutrient supplements	16 (46%)	9 (19%)	6 (13%)	8 (14%)
Folic acid supplements	35 (100%)	38 (97%)	0	0
Iron supplements	35 (100%)	47 (100%)	43 (90%)	4 (7%)

**Table 3:** Concentrations of B vitamins and metabolic markers in premature infants with very low birth weight from term to 12 months corrected age.

Parameters Median (IQR) Min, Max	Corrected Age			
	Term	2 Months	6 Months	12 Months
	*n* = 35	*n* = 47	*n* = 48	*n* = 58
Serum folate, nmol/L	118 (76, 137)	125 (93, 146)	41 (24, 58)	22 (18, 37)
	19, 200	7, 186	6, 140	4, 71
Serum cobalamin, pmol/L	392 (293, 531)	415 (301, 573)	448 (336, 638)	563 (428, 679)
	212, 662	69, 944	49, 1189	99, 1456
Plasma pyridoxal 5′-phosphate, nmol/L	177 (98, 300)	225 (93, 326)	176 (127, 225)	99 (73, 148)
	37, 434	23, 689	23, 484	22, 300
Plasma riboflavin, nmol/L	61 (27, 75)	42 (23, 66)	39 (30, 52)	39 (32, 47)
	18, 248	9, 276	17, 163	16, 138
Plasma homocysteine, µmol/L	5.9 (5.4, 6.7)	7.8 (6.5, 8.7)	5.9 (5.1, 7.6)	4.5 (4.1, 5.2)
	4.0, 10.2	3.6, 11.1	3.8, 10.7	3.1, 9.5
Plasma methylmalonic acid, µmol/L	0.54 (0.29, 1.53)	0.49 (0.26, 1.42)	0.32 (0.20, 0.61)	0.20 (0.14, 0.23)
	0.16, 9.01	0.11, 8.49	0.11, 2.14	0.09, 0.55

**Table 4:** Determinants of vitamin status in premature infants with very low birth weight at term and at 2, 6, and 12 months corrected age by multiple linear regression ^1^.

	Serum Folate, nmol/L		Serum Cobalamin, pmol/L		Plasma Pyridoxal 5′-Phosphate, nmol/L		Plasma Riboflavin, nmol/L		Plasma Total Homocysteine µmol/L		Plasma Methylmalonic Acid µmol/L	
	*B* ^1^	*p* Value	*B* ^1^	*p* Value	*B* ^1^	*p* Value	*B* ^1^	*p* Value	*B* ^1^	*p* Value	*B* ^1^	*p* Value
**At corrected age term**												
Human milk versus formula +/− human milk ^2^	−10	0.57	39	0.42	149	<0.001	49	0.01	1.1	0.04	−0.54	0.52
Infant vitamin supplementation ^3^	−8	0.66	42	0.40	84	0.02	50	0.02	0.5	0.33	−0.05	0.83
**At corrected age 2 months**												
Human milk versus formula +/− human milk ^2^	−31	0.02	177	0.002	171	<0.001	30	0.06	−0.7	0.15	−0.74	0.14
Infant vitamin supplementation ^3^	8	0.48	1	0.98	76	0.02	32	0.02	0.5	0.29	0.20	0.62
**At corrected age 6 months**												
Human milk versus formula +/− human milk ^2^	4	0.51	291	<0.001	70	0.003	18	0.03	−2.1	<0.001	−0.75	<0.001
Infant vitamin supplementation ^3^	17	0.23	−20	0.66	117	<0.001	7	0.26	0.3	0.29	0.10	0.25

The model additionally included gestational age, small for gestational age, weight at birth, corrected age, and maternal multivitamin supplementation. At 6 months, intake of solid food was included. ^1^ Unstandardized Beta (B); ^2^ human milk versus formula +/− human milk (number at term: 16/19, at 2 months: 17/30, at 6 months: 15/33); ^3^ number of infants with no supplement = 0, folic acid = 1, folic acid + multivitamins = 2 (at term: 0/20/15, at 2 months: 8/30/9, at 6 months: 0/0/6).

**Table 5:** Vitamin status in premature infants with very low birth weight in relation to exclusive breastfeeding versus non-exclusive breastfeeding and formula feeding from term to 6 months corrected age.

Parameters, Median (IQR)	Corrected Age								
	Term			2 Months			6 Months		
	Exclusive Breastfeeding	Formula +/− Human Milk	*p*^1^ Value	Exclusive Breastfeeding	Formula +/− Human Milk	*p* ^1^ Value	Exclusive Breastfeeding	Formula +/− Human Milk	*p*^1^ Value
	*n* = 16	*n* = 19		*n* = 17	*n* = 30		*n* = 15	*n* = 33	
Serum Folate, nmol/L	118 (68, 137)	113 (85, 137)	0.83	128 (113, 148)	115 (64, 141)	0.13	41 (17, 57)	41 (25, 64)	0.44
Serum Cobalamin, pmol/L	333 (246, 521)	475 (352, 531)	0.10	315 (201, 393)	519 (367, 605)	0.001	318 (189, 370)	541 (443, 657)	<0.001
Plasma Pyridoxal 5-phosphate, nmol/L	109 (79, 159)	291 (217, 399)	<0.001	93 (54, 140)	274 (175, 366)	<0.001	112 (82, 199)	196 (132, 261)	0.015
Plasma Riboflavin, nmol/L	32 (21, 41)	69 (62, 113)	<0.001	21 (16, 38)	55 (39, 71)	0.001	26 (19, 36)	46 (36, 56)	<0.001
Plasma Homocysteine, µmol/L	5.5 (4.7, 6.0)	6.4 (5.7, 8.3)	0.005	8.2 (7.1, 9.5)	7.4 (6.2, 8.3)	0.07	7.6 (6.6, 8.9)	5.4 (4.9, 6.4)	<0.001
Plasma Methylmalonic acid, µmol/L	0.57 (0.26, 1.67)	0.54 (0.29, 1.38)	0.83	1.06 (0.26, 3.03)	0.43 (0.26, 0.76)	0.74	0.91 (0.48, 1.33)	0.27 (0.19, 0.36)	<0.001

^1^ Mann–Whitney test.

## Data Availability

The dataset for the current study is available from the corresponding author upon reasonable request. Requests must justify the need for the data, ensuring it is for research purposes, while adhering to privacy, legal, or ethical restrictions that prevented immediate, open access.
